# Plasma metabolomic study in perinatally HIV-infected children using 1H NMR spectroscopy reveals perturbed metabolites that sustain during therapy

**DOI:** 10.1371/journal.pone.0238316

**Published:** 2020-08-31

**Authors:** Urvinder Kaur S., Bolaji Fatai Oyeyemi, Anita Shet, Bindu Parachalil Gopalan, Himanshu D., Neel Sarovar Bhavesh, Ravi Tandon

**Affiliations:** 1 Laboratory of AIDS Research and Immunology, School of Biotechnology, Jawaharlal Nehru University, New Delhi, India; 2 Transcription Regulation Group, International Centre for Genetic Engineering and Biotechnology (ICGEB), New Delhi, India; 3 International Vaccine Access Center, Johns Hopkins School of Public Health, Baltimore, MD, United States of America; 4 Division of Infectious Diseases, St. John’s Research Institute, St. John’s National Academy of health Sciences, Bangalore, India; 5 School of Integrative Health Sciences, University of Trans-Disciplinary Health Sciences and Technology (TDU), Bangalore, India; 6 Department of Medicine, King Georges Medical University, Lucknow, India; University of Nebraska Medical Center, UNITED STATES

## Abstract

**Background:**

Perinatally HIV-infected children on anti-retroviral treatment (ART) are reported to have metabolic abnormalities such as dyslipidemia, lipodystrophy, and insulin resistance which potentially increase the risk of diabetes, kidney, liver and cardiovascular disease.

**Objective:**

To elucidate HIV-mediated metabolic complications that sustain even during ART in perinatally HIV-infected children.

**Method:**

We have carried out metabolic profiling of the plasma of treatment-naïve and ART-suppressed perinatally HIV-infected children and uninfected controls using 1H nuclear magnetic resonance (NMR) spectroscopy followed by statistical analysis and annotation.

**Result:**

Validated multivariate analysis showed clear distinction among our study groups. Our results showed elevated levels of lactate, glucose, phosphoenolpyruvic acid, propionic acid, 2-ketobutyric acid and tricarboxylic acid (TCA) cycle metabolites in untreated HIV-infected children compared to uninfected controls. ART normalized the levels of several metabolites, however the level of lactate, phosphoenolpyruvic acid, oxoglutaric acid, oxaloacetic acid, myoinositol and glutamine remained upregulated despite ART in HIV-infected children. Pathway analysis revealed perturbed propanoate metabolism, amino acid metabolism, glycolysis and TCA cycle in untreated and ART-suppressed HIV-infected children.

**Conclusion:**

Developing therapeutic strategies targeting metabolic abnormalities may be beneficial for preventing diabetes, cardiovascular disease or other associated complications in perinatally HIV-infected children.

## Introduction

According to UNAIDS report 2019, there are approximately 1.7 million children less than 15 years old living with HIV worldwide and 160000 children became newly infected [1]. Maternal-to-child transmission (MTCT) accounts for more than 90% of pediatric HIV infection [2]. In the era of antiretroviral therapy (ART), the disease progression has been controlled and the life expectancy of HIV-infected children has greatly increased [3]. However, with an expanded life span, HIV-infected children have high prevalence of metabolic abnormalities such as dyslipidemia, lipodystrophy and insulin resistance, which potentially increases the risk of early cardiovascular atherosclerosis, kidney failure, and diabetes in this population [4, 5]. Several factors including HIV [6, 7], host immune response [8] and ART [4, 9] contribute to these metabolic complications.

Previous study demonstrated that prolonged exposure to protease inhibitors (PIs) in HIV-infected children was associated with lipid abnormalities and lipodystrophy [10]. The use of ART and persistent inflammation in children living with HIV were shown to be associated with increased carotid intima media thickness, which is a marker of cardiovascular disease (CVD) [11]. In HIV-infected children, increased use of ART also reported to cause dyslipidemia and insulin resistance [12, 13]. Thus, metabolic abnormalities in perinatally HIV-infected children are of great concern since ART is given early in life. However, even before the introduction of ART alterations in lipid metabolism and sensitivity to insulin were reported during HIV infection [14]. A recent study showed increased cardiovascular abnormalities in HIV-infected children without ART(treatment-naïve) [6]. Since existing clinical indicators of HIV are not infallible, observing the development of HIV infection in children and monitoring their responses to ART using NMR metabolic profiling will be germane in the identification of novel biological markers. Several studies had focused on the impact of ART on metabolism in perinatally HIV-infected children [3, 4, 6, 15, 16], metabolic complications due to HIV in treatment-naïve HIV-infected children is less understood. It is thus imperative to monitor metabolic state of children with or without ART.

Metabolomics is an unbiased method to identity and quantify metabolites present in the biological fluids and tissues, providing the metabolic fingerprint of an organism. Metabolomics helps to determine metabolic alterations during various diseases like coronary heart disease [17], colon cancer [18] and hepatitis [19] and has been used to discover novel clinical biomarkers and therapeutic targets.

The NMR spectroscopy was first used by Hewer et al. to profile metabolites in the serum of chronically HIV-infected adults. They demonstrated a distinction in the sera among treatment-naïve, ART-suppressed (ART-experienced and virologically suppressed) HIV-infected subjects and HIV negative controls [20]. Since then, other studies have used NMR and MS to demystify metabolic response to HIV in adults [21–23]. Moreover, it is also necessary to comprehensively study HIV-infected children plasma metabolic profile as they have remarkable difference in HIV-disease progression with higher viremia compared to HIV-infected adults [24, 25] and also have metabolic abnormalities [4, 5]. In this study, we performed untargeted metabolic profiling of treatment-naïve and ART-suppressed perinatally HIV-infected children and compared them to age-matched uninfected controls by using 1H NMR spectroscopy.

## Material and methods

### Study participants

This pilot cross-sectional study included 30 perinatally HIV-infected children and 12 age-matched uninfected controls who had been recruited in June 2016 from the south part of India. All the children were less than 15 years old. Perinatal infection was confirmed by the documentation of HIV infection in children within 1 year of life along with documentation of maternal HIV infection. Among HIV-infected children, 15 children were on ART for more than 6 months, whereas the other 15 children were not on ART (treatment-naïve). Those on ART had undetectable plasma viral load (VL < 50 copies/ml) and 60% of them were receiving a combination of Zidovudine (ZDV), Lamivudine (3TC) plus Nevirapine (NVP), 20% were receiving Atazanavir (ATV), 3TC plus Efavirenz (EFV), 13% were receiving Stavudine (d4T), 3TC plus NVP and the remaining 7% were receiving ZDV, 3TC plus Lopinavir/Ritonavir (LPV/r). Uninfected controls were matched with the HIV-infected children for age and sex. Children with age greater than 15, with co-infections such as tuberculosis or viral hepatitis and metabolic abnormalities were excluded from this study. All study participants belonged to same ethnicity, geographical area and had similar dietary habits. This study was approved by Institutional Review Board (IRB) of St. Johns Medical College & Hospital at Bangalore, Jawaharlal Nehru University (JNU) at New Delhi and King George’s Medical University (KGMU) Lucknow, and was carried out in accordance with the approved guidelines. The biosafety approval was obtained from Institutional Biosafety Committee (IBSC) of JNU for handling plasma samples. Informed written consent was obtained from parents/guardian of all the children who participated in our study. The characteristics of subjects are listed in Table 1.

**Table 1:** Subjects characteristics.

Parameters	Treatment naïve HIV-infected children	ART-suppressed HIV- infected children	HIV negative controls
**Number**	15	15	12
**Age (years)**	10 (8–12)	11 (9–11)	10 (8–11)
**CD4 count (cells per ul)**	615 (423–749)	1157(594–1336)	1338 (1112–1449)
**Plasma viral load (HIV RNA copies per ml)**	28,410 (10,842–60,193)	< 50	NA
**ART regimens**	NA	ZDV+3TC+NVP (60%)	NA
		ATV+3TC+EFV (20%)	
		d4T+ 3TC+ NVP (13%)	
		ZDV+ 3TC+ LPV/r (7%)	

Values are shown as median (IQR), NA: Not applicable.

#### Plasma viral load and CD4 count

Plasma HIV-1 viral load (VL) was measured using Abbott Real Time HIV-1 assay with a lower limit of detection of 50 copies of RNA/ml (Abbott Molecular Inc., Des Plaines, IL, USA). CD4 T cell count was measured using FC500™ flow cytometer (Beckman Coulter, Fullerton, California, USA).

#### Sample collection

Peripheral blood was collected into vacutainer blood collection tubes containing EDTA and whole blood was centrifuged to isolate plasma. Plasma samples were stored at -80°C for NMR metabolic profiling.

### Sample preparation and NMR spectroscopy

Plasma samples were thawed on ice and centrifuged at 10,000g for 10 min to remove particulates. 70 μL of supernatant was then taken and immediately added to 140 μL of 0.1M potassium phosphate buffer (composition: 0.2M of K_2_HPO_4_ and KH_2_PO_4_, 10% 4,4-dimethyl-4-silapentane-1-sulfonic acid (DSS), D_2_O, pH 7.4). The mixture was transferred to an NMR tubes (outer diameter: 3.0 mm) for NMR analysis. NMR experiments were performed at 298K on a Bruker 500.13 MHz AVANCE III spectrometer equipped with 5mm triple-resonance z-gradient cryoprobe (CPTCI 1H–31P/13C/2D Z-GRD). TopSpin, version 3.5 (Bruker Corporation), was used for spectrometer control. Temperature was calibrated using a 100% d4-methanol sample. ^1^H Carr–Purcell–Meiboom–Gill (CPMG) echo sequence was used to record NMR spectra. A total CPMG delay of 300 ms was used with an echo time of 200 μs was used based on experimental optimization. NMR spectra were obtained with 32 scans and a relaxation delay of 4 sec. Free induction decays (FIDs) 10,000 Hz spectral width and 3.33 sec acquisition time were collected for each sample.

### NMR data processing

NMR data processing was done using Topspin, version 3.5. Automatic matching, tuning and shimming of each data were done. They were Fourier transformed after zero-filling to varying points and application of a line-broadening factor of 0.30 Hz. Consequently, NMR spectra were phased and automatically baseline corrected. Spectra were referenced to DSS at 0 ppm and spectral region between 4.5 and 4.7 ppm was set to zero to remove water resonance suppression effect. Peaks for each samples were picked and each NMR peak list and intensity data were then imputed into Metaboanalyst 3.0. This program groups peaks based on their ppm values using a moving window of 0.03 ppm and a step of 0.015 ppm and peaks within the same group were aligned to their median ppm. Peaks appearing in less than half of the samples were excluded from downstream analysis.

### Statistical analysis, metabolite identification and pathway analysis

MetaboAnalyst 3.0 (http://www.metaboanalyst.ca/) was used for statistical analyses and NMR spectral annotations [26]. This is a web server with numerous statistical and machine learning algorithms for comprehensive metabolomics data analysis, visualization, and interpretation. Processed raw data from TopSpin were log transformed, normalized by sample median and auto-scaled by mean-centering and dividing by the standard deviation of each variable for better, reliable and accurate biological inference before univariate and multivariate data analysis in Metaboanalyst 3.0 [27].

Supervised and unsupervised multivariate statistical analysis including principal component analysis (PCA), partial least squares-discriminant analysis (PLS-DA) and orthogonal partial least squares-discriminant analysis (OPLS-DA) were performed to discriminate between our study groups. Metabolites responsible for difference in the metabolic profiling were obtained from variable importance in projection plot (VIP) of threshold 1.0 in PLS-DA. Both PLS-DA and OPLS-DA models were validated for predictability and PLSDA: R^2^, accuracy, Q^2^ and OPLS-DA: R^2^Y and Q^2^ were generated to confirm whether the difference between our study groups was statistically significant. One-way analysis of variance (ANOVA) was employed to analyze the significance of differential metabolites across our study groups.

Corresponding metabolites for differential peaks were identified from NMR spectra databases which include Human Metabolome Data Bank (HMDB) [28] and complex mixture analysis (COLMAR) query web server [29] as listed in Table 2. These metabolites were checked with two-dimensional (2D) homonuclear ^1^H-^1^H J-resolved experiments and compared with previously published one and two dimentional values [18, 20, 22]. The representative 500 MHz 1H-1H J-Resolved (JRes) spectrum is shown in S1 Fig. Metabolic pathway analysis was carried out using MetaboAnalyst 3.0 [26], Kyoto Encyclopedia of genes and genomes (KEGG) [30] and HMDB pathway.

**Table 2:** Nuclear magnetic resonance chemical shift assignments of differential compounds identified in serum of treatment naïve, ART-suppressed and uninfected control.

S/N	Compound	HMDB ID	Chemical shift (ppm)^a^
1	Leucine	HMDB0000687	0.935 (d), 0.947 (d), 1.633 (m), 1.700 (m)
2	Valine	HMDB0000883	0.97 (d), 1.02 (d), 2.23 (m)
3	Propionic acid	HMDB0000237	1.043 (t), 2.170 (q)
4	2-Ketobutyric acid	HMDB0000005	1.069 (t), 2.760 (q)
5	Ethanol	HMDB0000108	1.170 (t), 3.647 (q)
6	β-hydroxybutyrate	HMDB0000011	1.204 (d), 2.314 (dd), 2.414 (dd), 4.160 (m)
7	Caproic acid	HMDB0000535	0.864 (t), 1.274 (m), 1.536 (m), 2.518 (t)
8	Lactate	HMDB0000190	1.32 (d), 4.10 (q)
9	Dimethylmalonic acid	HMDB0002001	1.430 (s)
10	Alanine	HMDB0000161	1.46 (d) 3.77 (q)
11	2-Ethyl-2-Hydroxybutyric acid	HMDB0001975	0.873 (t), 1.670 (m), 1.795 (m)
12	Leucine	HMDB0000687	0.935 (d), 0.947 (d), 1.633 (m), 1.700 (m)
13	Ethylmalonic acid	HMDB0000622	0.889 (t), 1.744 (m), 3.023 (t)
14	Putrescine	HMDB0001414	1.754 (m), 3.040 (t)
15	N6-acetyl-L-lysine	HMDB0000206	1.409 (m), 1.562 (m), 1.871 (m), 1.990 (s), 3.195 (q), 3.740 (t)
16	Acetate	HMDB0000042	1.91 (s)
17	N-acetyl-L-alanine	HMDB0000766	1.315 (d), 2.000 (s), 4.114 (t)
18	Methionine	HMDB0000696	2.12 (s), 2.61 (t)
19	Suberic acid	HMDB0000893	1.296 (s), 1.538 (s), 2.163 (s)
20	Acetone	HMDB0001659	2.22 (s)
21	Acetoacetate	HMDB00060	2.29 (s)
22	Oxaloacetic acid	HMDB0000223	2.377 (s)
23	Succinic acid	HMDB0000254	2.393 (s)
24	Glutamine	HMDB0000641	2.06 (m), 2.38 (m), 3.65 (t)
25	Pyridoxal	HMDB0001545	2.448 (s), 5.038 (d), 5.219 (d), 6.542 (d), 2.448 (s)
26	Citrate	HMDB0000094	2.52 (d), 2.67(d)
27	2,2-Dimethylsuccinic acid	HMDB0002074	1.250 (s), 2.660 (s)
28	Sarcosine	HMDB0000271	2.72 (s), 3.60 (s)
29	Aspartate	HMDB0000191	2.62 (dd), 2.78 (dd), 3.86 (t)
30	Dimethylglycine	HMDB0000092	2.910 (s), 3.710 (s)
31	Oxoglutaric Acid	HMDB0000208	2.428 (m), 2.996 (m)
32	Creatine	HMDB0000064	3.02 (s), 3.92 (s)
33	Ethanolamine	HMDB0000149	3.130 (d), 3.810 (d)
34	Choline	HMDB0000097	3.19 (s)
35	Glycerophosphorylcholine	HMDB0000086	3.200 (s), 3.637 (m), 3.897 (m), 4.305 (m)
36	Trimethylamine N‐oxide	HMDB0000043	3.25 (s), 3.89 (s)
37	1,3-Dimethyluric acid	HMDB0001857	3.298 (s), 3.428 (s)
38	Theophylline	HMDB0001889	3.340 (s), 3.535 (s) 7.990 (s)
39	Methylguanidine	HMDB0001522	2.833 (s), 3.366 (s)
40	Taurine	HMDB0000251	3.19 (t), 3.41 (t)
41	Cis-Aconitic acid	HMDB0000072	3.435 (d). 6.580 (s)
42	β−Glucose	HMDB0000122	3.39 (m), 3.46 (m), 3.48 (m), 3.71 (m), 3.89 (m), 4.64 (d)
43	Glycine	HMDB0000123	3.52 (s)
44	Myo-inositol	HMDB0000211	3.54 (dd), 3.63 (t), 4.06 (m)
45	Glucono-1,5-lactone	HMDB0000150	3.666 (dd), 3.767 (m), 3.817 (dd), 4.027 (d), 4.125 (d)
46	N-Methyl-a-aminoisobutyric acid	HMDB0002141	1.590 (s), 3.840 (s)
47	Mannitol	HMDB0000765	3.69 (m), 3.88 (m)
48	Serine	HMDB0000187	3.91 (m), 3.77 (m)
49	Isopropyl alcohol	HMDB0000863	1.17 (d), 3.98 (m)
50	6-Phosphogluconic acid	HMDB0001316	3.840 (m), 3.961 (m), 4.095 (m), 4.185 (d)
51	Fructose	HMDB0000660	3.577 (m), 3.695 (m), 3.823 (m), 3.903 (dd), 4.005 (m), 4.029 (dd), 4.118 (m)
52	Glyceric acid	HMDB0000139	3.779 (m), 4.133 (m)
53	Pyridoxamine 5'-phosphate	HMDB0001555	2.461 (s), 4.314 (s), 4.852 (s), 7.645 (s)
54	2,4-Diamino-6hydroxypyrimidine	HMDB0002128	4.953 (s)
55	Phosphoenolpyruvic acid	HMDB0000263	5.180 (t), 5.360 (t)
56	3,4-Dihydroxymandelic acid	HMDB0001866	6.843 (dd), 6.888 (d), 6.918 (d)
57	Tyrosine	HMDB0000158	7.17 (d), 6.86 (d), 2.98 (q)
58	2-Furoylglycine	HMDB0000439	3.935 (d), 6.643 (dd), 7.190 (d), 7.700 (d)
59	Indoxyl sulfate	HMDB0000682	7.209 (dd), 7.281 (dd), 7.362 (s), 7.508 (d), 7.712 (d)
60	4-Pyridoxic acid	HMDB0000017	2.310 (s), 4.510 (s), 7.530 (s)
61	Mandelic acid	HMDB0000703	4.980 (s), 7.380 (m), 7.420 (d)
62	Phenylacetylglycine	HMDB0000821	3.665 (s), 3.744 (d), 7.380 (m)
63	Quinolinic acid	HMDB0000232	7.440 (q), 7.990 (t), 8.430 (t)
64	Formic acid	HMDB0000142	8.440 (s)

^a^Multiplicity: s singlet, d doublet, t triplet, q quartet, dd doublet of doublets, m multiplet.

## Results

### Multivariate analysis distinguishes plasma metabolic profile among treatment-naïve, ART-suppressed perinatally HIV-infected children and uninfected controls

**Fig 1:**
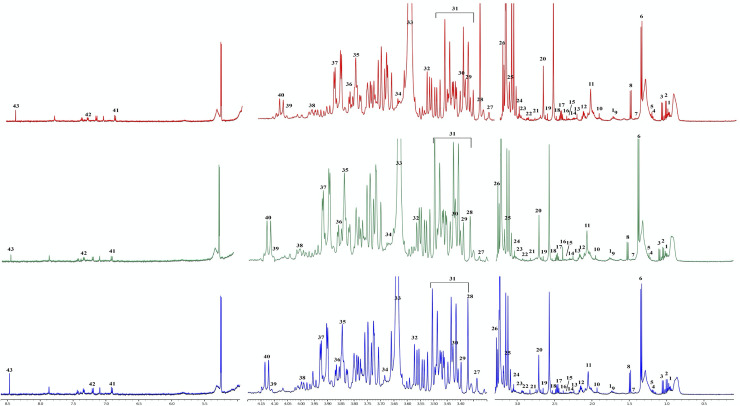
Representative 500‐MHz 1H nuclear magnetic resonance spectra of serum from treatment naïve (green), ART-suppressed (green) perinatally HIV-infected child and uninfected control (blue). 1‐ Leucine, 2- valine, 3–2-Ketobutyric acid, 4- ethanol, 5- β-hydroxybutyrate, 6- lactate, 7- dimethylmalonic acid, 8- alanine, 9–2-ethyl-2-Hydroxybutyric acid, 10- acetate, 11- n-acetyl-L-alanine, 12- methionine, 13- acetone, 14- acetoacetate, 15- oxalacetic acid, 16- succinic acid, 17- glutamine, 18- pyridoxal, 19- citrate, 20- sarcosine, 21- aspartate, 22- dimethylglycine, 23- oxoglutaric acid, 24- creatine, 25- choline, 26- trimetylamine N‐oxide, 27- theophylline, 28- methylguanidine, 29- taurine, 30- cis-aconitic acid, 31- β−glucose, 32- glycine, 33- myo-inositol, 34- glucono-1,5-lactone, 35- N-methyl-a-aminoisobutyric acid, 36- mannitol, 37- serine, 38- isopropyl alcohol, 39- fructose, 40- glyceric acid, 41- tyrosine, 42–2-furoylglycine, 43- formic acid. Broken lines indicate excluded spectral region.

A representative one dimensional ^1^H-NMR spectra of plasma from treatment-naïve, ART-suppressed perinatally HIV-infected child and uninfected control is depicted in Fig 1.

**Fig 2:**
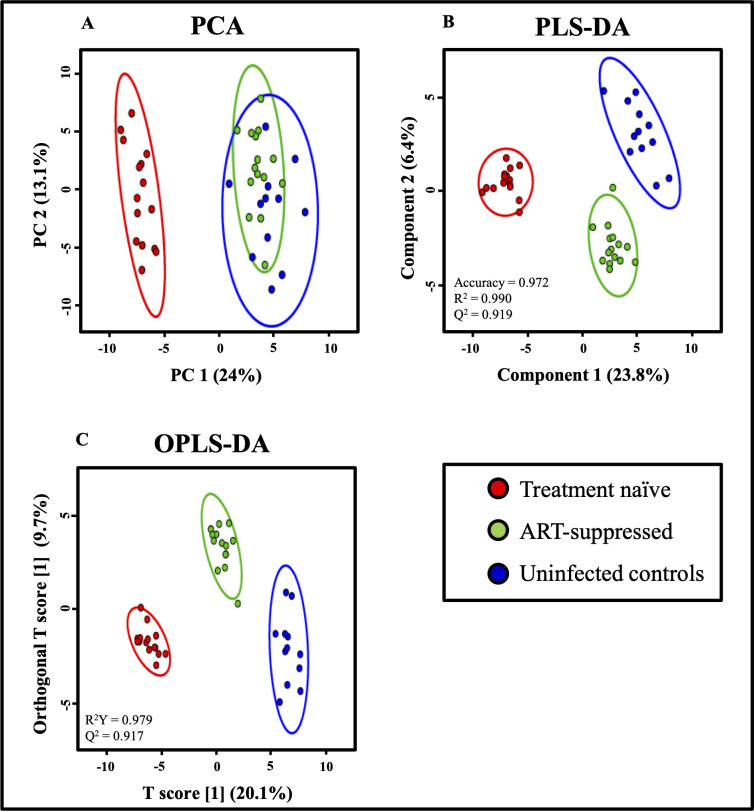
A. PCA B. PLS-DA C. OPLS-DA score plots developed from 1H NMR spectra of plasma of treatment-naïve (red), ART-suppressed (green), perinatally HIV-infected children and uninfected controls (blue). Each dot represents individual samples.

To determine whether it was possible to differentiate HIV-infected children from uninfected controls on the basis of the NMR spectra, we carried out unsupervised multivariate analysis such as PCA and supervised multivariate analysis such as PLS-DA and OPLS-DA. PCA score plot showed that treatment-naïve HIV-infected children could be distinguished from ART-suppressed HIV-infected children and uninfected controls (Fig 2A). Whereas PLS-DA score plot revealed excellent separation among treatment-naïve, ART-suppressed perinatally HIV-infected children and controls (Fig 2B and 2C). On validation by permutation tests, we obtained PLS-DA model with accuracy 0.972 (R^2^ = 0.990 and Q^2^ = 0.919) and p = 0.02 and OPLS-DA with R^2^Y = 0.979 and Q^2^ = 0.917. For biological data, a model with R^2^ = 0.7 and Q^2^ = 0.4 is considered to be good.

### Perturbed levels of metabolites in HIV-infected children despite suppressive therapy

**Fig 3:**
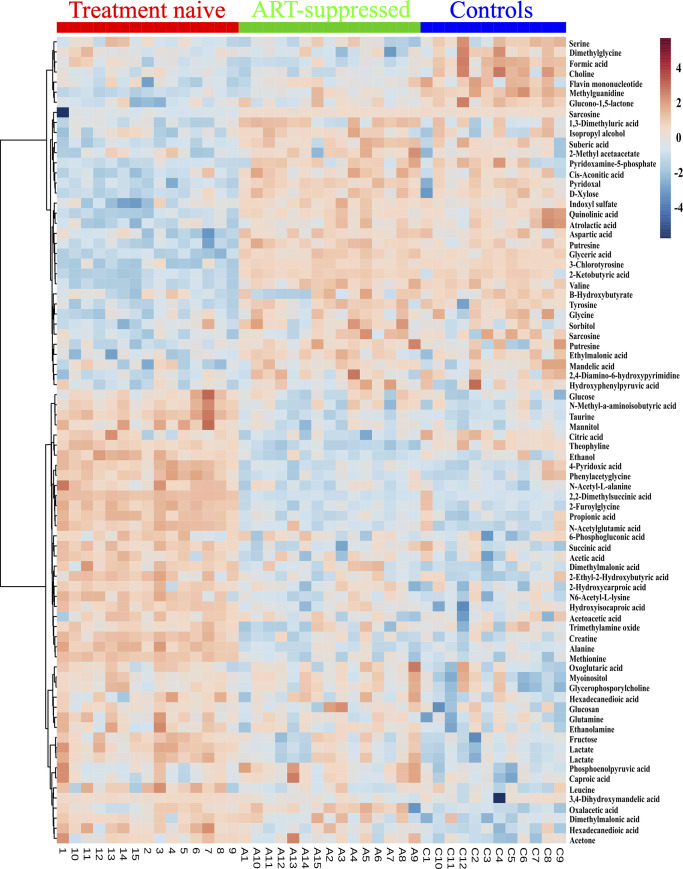
Unsupervised hierarchical clustering of top 75 metabolites that distinguish treatment-naïve, ART-suppressed perinatally HIV infected children from uninfected controls. Each cell in the heat map denotes relative level of metabolites, with samples in column and metabolites in the row. Red and blue indicate increased and decreased levels, respectively.

To identify the metabolites with the highest degree of differences in the metabolomes among treatment-naïve, ART-suppressed HIV-infected children and uninfected controls, we used PLS-DA to evaluate variable importance in projection (VIP) scores. A heatmap showing relative intensity of some metabolites as identified by VIP scores (Fig 3). We identified 66 metabolites to be significantly altered in untreated HIV-infected children when compared to uninfected controls. Out of these, the levels of 38 metabolites were significantly high in untreated HIV-infected children compared to controls, while 28 metabolites were significantly low in untreated HIV-infected children compared to controls (S1 Table).

**Fig 4:**
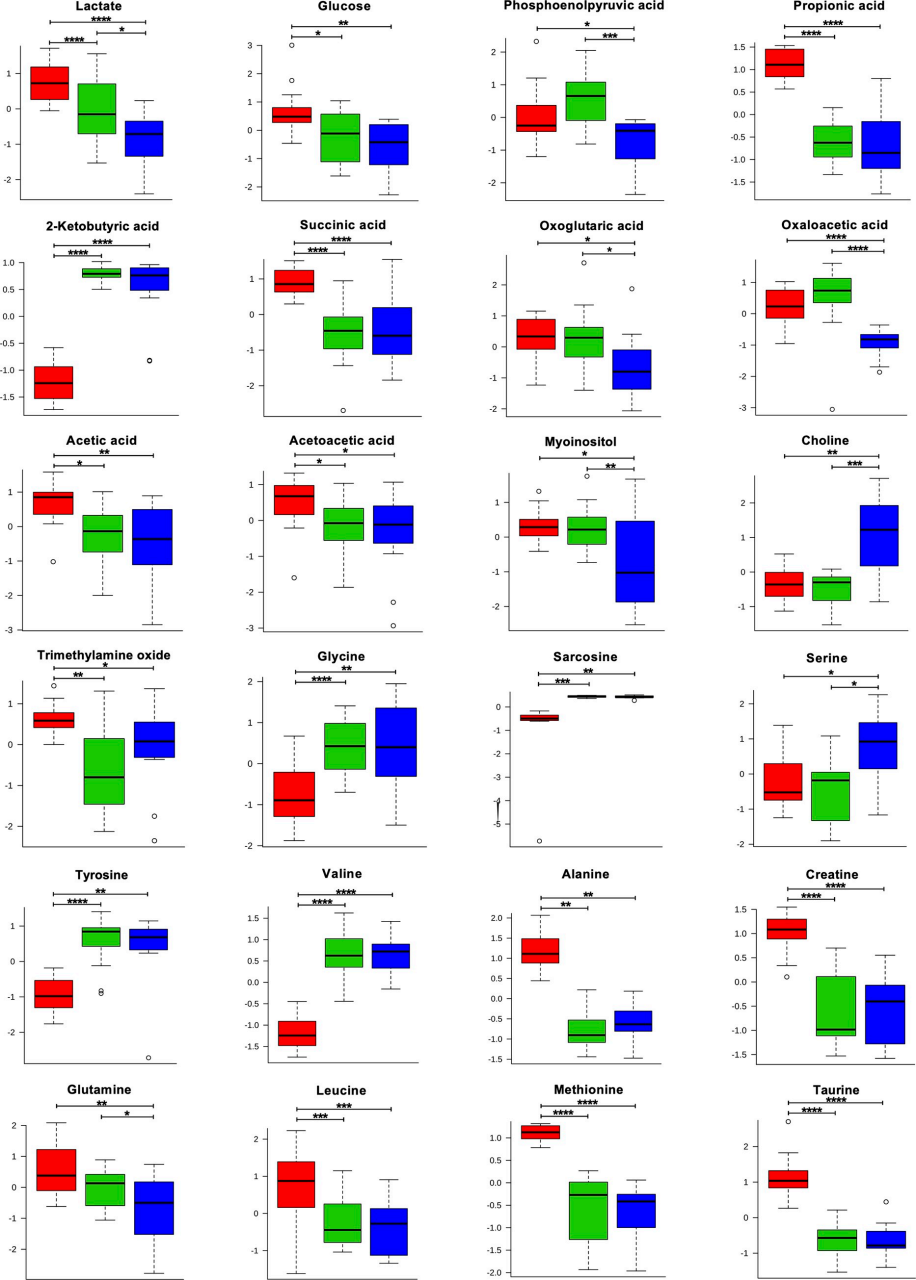
Box plots illustrating the levels of representatives metabolites in the plasma of treatment-naïve (red), ART-suppressed (green) perinatally HIV infected subjects and uninfected controls (blue). The y-axis shows the normalized concentrations of the metabolites. Each box represents interquartile range (IQR) between 25% and the 75% percentiles while the error bar represents 5% and 95% percentiles. Horizontal line within box denotes the median value. Circles indicate the single data points. * = p < 0.05, ** = p<0.005, *** = p <0.00005, **** = p >0.00005. p < 0.05 was statistically significant.

Insulin resistance is one of the major metabolic disorders seen in patients living with HIV [31]. Within altered metabolites, the levels of lactate, glucose, phosphoenol pyruvic acid and TCA cycle metabolites such as succinic acid, oxoglutaric acid, and oxaloacetic acid were significantly higher in the plasma of treatment-naïve HIV-infected children compared to uninfected controls. Compared to controls, the plasma levels of some other metabolites like propionic acid, acetate, acetoacetate, myoinositol and trimethylamine-N-oxide (TMAO) were significantly elevated whereas 2-ketobutyric acid and choline were siginificantly lowered in treatment-naïve HIV-infected children. treatment-naive HIV-infected children also showed abnormalities at the aminoacid levels with significantly lower plasma level of glycine, sarcosine, serine, tyrosine, valine and higher plasma level of alanine, creatine, glutamine, leucine, methionine and taurine compared to controls (Fig 4 and S1 Table).

In treated children the plasma levels of glucose, propionic acid, 2-ketobutyric acid, succinic acid, acetic acid, acetoacetic acid, TMAO, glycine, sarcosine, tyrosine, valine, alanine, creatine, leucine, methionine, and taurine were restored to the levels found in uninfected controls. However, the plasma levels of lactate, phosphoenolpyruvic acid, oxoglutaric acid, oxaloacetic acid, myoinositol and glutamine remained elevated and the plasma levels of choline and serine remained lowered in treated HIV-infected children suggesting the failure of ART in restoring the levels of these metabolites (Fig 4 and S1 Table).

### Metabolic pathway analysis

**Fig 5:**
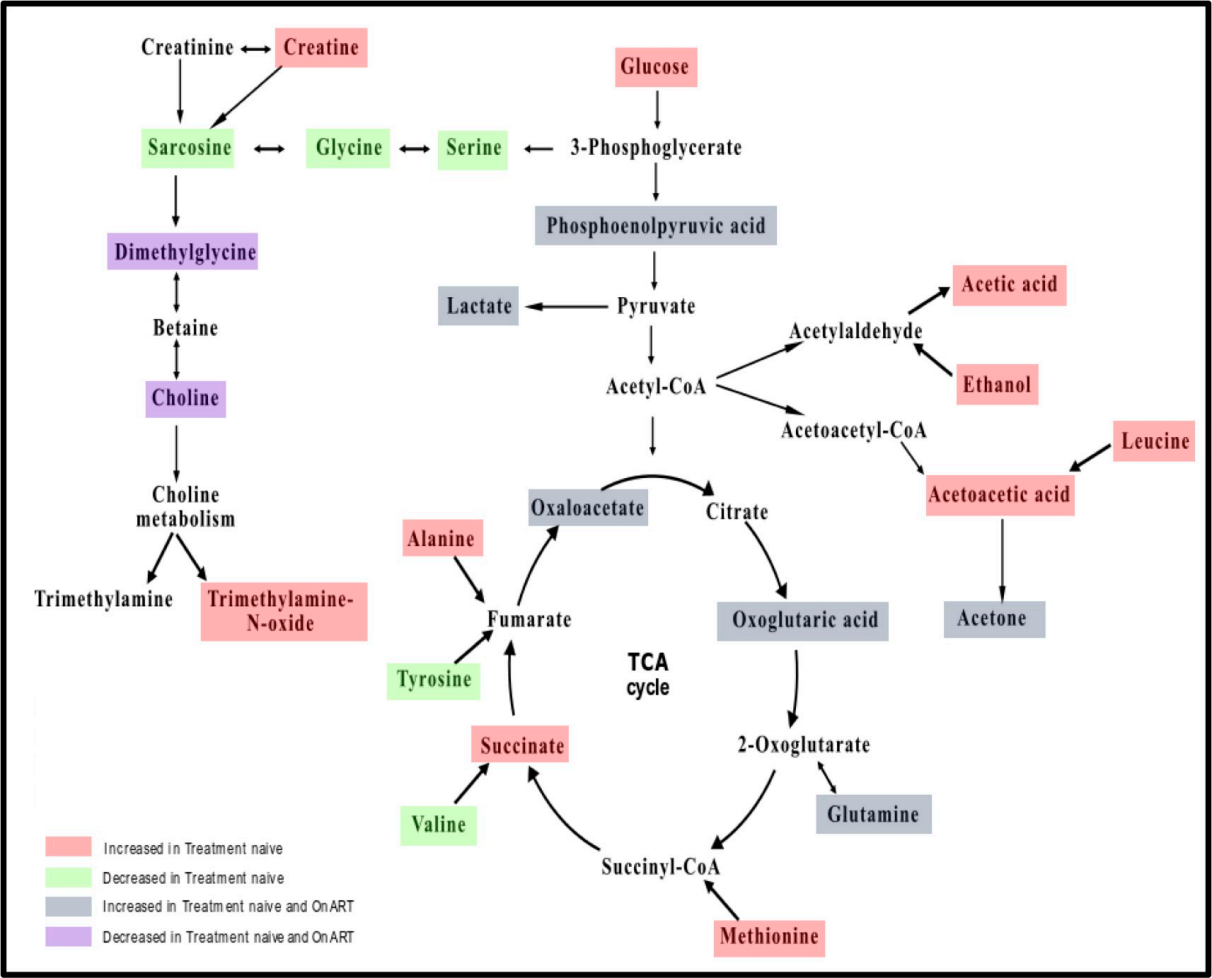
Perturbed metabolic pathways in treatment-naïve and ART-suppressed perinatally HIV infected children.

Metabolites that were significantly altered in treatment-naïve and ART-suppressed perinatally HIV-infected children when compared to controls, were subjected to pathway analysis. We found 22 metabolic pathways among treatment-naïve, ART-suppressed perinatally HIV-infected children and uninfected controls that were significantly modulated (S2 Table). The prominent changes were observed in amino acid metabolism, TCA cycle, glycolysis pathway (Fig 5).

## Discussion

In this study, we profiled the plasma metabolome of treatment-naïve and ART-suppressed HIV-infected children, and compared them with that of uninfected controls using NMR spectroscopy. Our data suggests perturbed levels of metabolites in treatment-naïve perinatally HIV-infected children compared to uninfected controls. The ART appears to normalize majority of metabolites in perinatally HIV-infected children under treatment; some metabolites like lactate, phosphoenolpyruvic acid, oxoglutaric acid, oxaloacetic acid, myoinositol and glutamine remain elevated either due to previous exposure to HIV or ART itself.

Altered metabolites due to perturbed metabolic process might cause metabolic disorders like insulin resistance, hyperlipidemia, obesity and high blood pressure. It was beyond the scope of clinical routine practice and the study scope to measure lipid levels, insulin resistance etc. However, none of the HIV-infected children in this study had high BMI. Previous studies have shown a direct link between perturbed glycolytic pathway and chronic inflammation [32, 33]. Metabolic profiling of plasma of HIV-infected adults had shown higher levels of glucose and lactic acid, which is suggestive of perturbed glycolytic pathway [22]. In our study, the increase in the level of glucose, lactic acid and phosphoenolpyuvic acid among treatment-naïve perinatally HIV-infected children is an indication of perturbed glycolysis and supports the previous findings in HIV-infected adults [22]. In addition, high level of glucose or hyperglycemia has been known to promote HIV pathogenesis. This was supported by a study which showed that the high glucose increases the expression of CXCR4 in T cells thereby enhancing the entry of HIV into T cells [34]. Glucose metabolism has also been reported to play a major role in interleukin-1β (IL-1β) production [35] and thus high glucose level during chronic HIV infection results in the upregulation of IL-1β level in the plasma of HIV-infected subjects. A study by Mikulak et al showed that IL-1β enhances HIV entry and its persistence in human podocytes [36].

Interestingly, the use of ART does not seem to have any effect on the perturbed glycolytic pathway. In ART-suppressed HIV-infected children, the glucose level is restored to that of uninfected controls, however, the levels of lactate and phosphoenolpyruvic acid remain elevated despite the suppressive therapy. Hyperlactatemia has been reported in HIV-infected children receiving antiretroviral treatment as well as in ART-exposed HIV uninfected children born to HIV-infected mothers [37, 38]. High plasma lactate concentration is directly associated with insulin resistance and increased hepatic gluconeogenesis in type-2 diabetic subjects [39]. Our results of high levels of lactate in treated and untreated perinatally HIV-infected children are in agreement with the previous finding [37, 38, 40] and suggests that perinatally HIV-infected children are at high risk of developing insulin resistance and diabetes even during suppressive treatment.

High level of plasma succinic acid is associated with inflammation as shown in case of cancer [41]. It induces primary inflammatory cytokine, IL-1β, through HIF-1α signaling [42]. Also, when dendritic cells (DCs) were simultaneously primed with both succinate and antigen, T cell activation was increased, as shown by elevated TNF-α and IFN-γ production from these cells [43]. Thus, succinate is an important metabolite that can act as an inflammatory signature. Increased levels of succinic acid in treatment-naïve perinatally HIV-infection children reflect elevated inflammation that appears to normalize during treatment.

We observed elevated levels of plasma TMAO and propionic acid, a gut-microbiome dependent metabolite, in untreated HIV-infected children. Gut microbiota has a significant role in metabolism of host. Gut microbiota is associated with numerous metabolic conditions including diabetes, obesity, and CVD [44, 45]. Choline which comes from dietary phosphatidylcholine is metabolized by gut microbes to trimethylamine (TMA), which further oxidizes to TMAO in liver. Wang et al. showed that the metabolites of dietary phosphatidylcholine promote cardiovascular disease (CVD) [46]. Plasma level of TMAO has been shown to be associated with CVD in general population [47, 48] and carotid artery atherosclerosis progression in HIV-infected adults [49]. This suggests that perinatally HIV-infected children are at the risk of developing CVD. Further, propionic acid, that is synthesized by the gut microbes during complex carbohydrates fermentation in the colon is taken up to the circulation where it further metabolizes in the liver and serves as an energy source. Propionic acid regulates glucose homeostasis and is antiobesogenic [50]. However, elevated levels of propionic acid were reported in obese individuals [51] and also in obese diabetes mice [52], indicating that propionic acid might may pathogenic roles via mechanisms yet to be elucidated. The increase in TMAO and propionic acid level in untreated HIV-infected children might be attributed to the gut dysbiosis as we observed in our previous study [53].

Elevated Myo-inositol among untreated HIV-infected children is similar to earlier reports where they showed myo-inositol to be elevated in left frontal brain of HIV-infected children [54]. Increased myo-inositol in both gray and white matter and also in basal ganglia regions were reported in chronically HIV-infected adults [55, 56]. Myo-inositol is a marker of glial cells and elevated myo-inositol indicates gliosis, a condition of proliferation or hypertrophy of glial cells in response to damage to the central nervous system (CNS). Increased myo-inositol in both treatment-naïve and ART-suppressed HIV-infected children suggests the presence of neuroinflammation and thus might potentially contribute to neurocognitive disorders despite successful virologic control by ART. Previous study has also demonstrated neuroinflammation and cognitive impairment in treated HIV-infected adults [57].

Increased glutamine among untreated HIV-infected children could reflect CD4 T cell skewing and activation and as shown earlier in mice [58]. This was further confirmed by Wang et. al, where glutamine catabolism was shown to be tightly coupled with the biosynthesis of polyamines that are essential for T cell proliferation [59].

A study by Ziegler et al reported decreased levels of plasma amino acids except glutamate in HIV-infected youth [60]. Our study also observed decreased valine, aspartatic acid, glycine, tyrosine, serine and sarcosine levels in the HIV infected treatment-naïve group. This might be due to intestinal malabsorption as observed in HIV-infected individuals. Intestinal epithelial apoptosis and increased epithelial barrier disruption during chronic HIV infection [61] might reduce amino acids absorption. In addition to nutritional deficit, the change in plasma amino acids levels is also due to alteration in aminoacid metabolic pathway.

In conclusion, our NMR-based metabolomics with an array of robust multivariate statistical techniques have unraveled distinct metabolic phenotypes and pathways among treatment-naïve, ART-suppressed perinatally HIV-infected children and uninfected controls. Untreated and ART-suppressed HIV-infected children had perturbed glycolysis, TCA cycle, aminoacid metabolism. Developing therapeutic stratergies targeting metabolic abnormalities and restoring healthy gut microbiota may be beneficial for preventing diabetes, cardiovascular disease or other associated complication in perinatally HIV-infected children.

## Supporting information

S1 Fig The representative 500 MHz 1H-1H J-Resolved (JRes) spectrum. Three different spectral region of the 1D 1H Jres spectrum are expanded for improved visualization: (A) *δ* (0.0–3.0) ppm, (B) *δ* (3.0–4.10) ppm and (C) *δ* (4.80–9.00) ppm. Assigned peaks are annotated.
(SVG)

S1 Table Significantly altered metabolites in treatment naïve and ART-suppressed HIV infected children. (PDF)

S2 Table Perturbed metabolic pathways between treatment naïve, ART-suppressed HIV infected children and uninfected controls. (PDF)

S1 Raw data Compressed file containing NMR raw data of all study subjects. (TGZ)
