# Supplementary material for: Plasma metabolomic study in perinatally HIV-infected children using 1H NMR spectroscopy reveals perturbed metabolites that sustain during therapy
Source: PLoS One. 2020 Aug 31;15(8):e0238316. doi: 10.1371/journal.pone.0238316 (PMC7458310; doi:10.1371/journal.pone.0238316)
Supplement: S1 Table — (PDF) [file pone.0238316.s002.pdf]

S1 Table. Significantly altered metabolites in treatment naïve and ART-suppressed HIV infected children.

Metabolites	Ppm	Treatment naïve vs controls	ART-suppressed vs controls	Treatment naïve vs ART-suppressed
1,3-Dimethyluric acid	3.28	NS	High	Low
2,2-Dimethylsuccinic acid	2.68	High	Low	High
2,4-Diamino-6-hydroxypyrimidine	4.94	Low	NS	Low
2-Ethyl-2-Hydroxybutyric acid	1.67	High	NS	High
2-Furoylglycine	7.18	High	NS	High
2-Ketobutyric acid	1.06	Low	NS	Low
2-Methyl acetoacetate	2.29	NS	NS	Low
3,4-Dihydroxymandelic Acid	6.87	High	NS	NS
3-Chlorotyrosine	1.49	Low	NS	Low
4-Pyridoxic acid	7.32	High	NS	High
6-Phosphogluconic acid	4.08	High	NS	NS
Acetic acid	1.92	High	NS	High
Acetoacetic acid	2.26	High	NS	High
Acetone	2.23	High	High	NS
Alanine	1.47	High	NS	High
Aspartic Acid	2.82	Low	NS	NS
Atrolactic acid	7.35	Low	NS	Low
β-hydroxybutyrate	1.19	Low	NS	NS
Caproic acid	1.28	NS	High	NS
Choline	3.17	Low	Low	NS
cis-Aconitic acid	3.43	Low	NS	Low

Citric acid	2.64	NS	Low	High
Creatine	3.02	High	NS	High
Dimethylglycine	2.92	Low	Low	NS
Dimethylmalonic acid	1.41	High	High	High
D-Xylose	3.50	Low	NS	Low
Ethanol	1.17	High	Low	High
Ethanolamine	3.14	High	NS	NS
Ethylmalonic acid	1.74	Low	NS	Low
Flavin mononucleotide	2.51	Low	Low	Low
Formic acid	8.46	NS	Low	NS
Fructose	4.11	High	NS	High
Glucono-1,5-lactone	3.66	Low	Low	Low
Glucosan	5.44	High	NS	NS
Glucose	3.48	High	NS	High
Glutamine	2.44	High	High	NS
Glyceric acid	4.14	Low	NS	Low
Glycerophosphorylcholine	3.23	High	High	NS
Glycine	3.55	Low	NS	Low
Hexadecanedioic acid	0.70	High	High	High
Hydroxyisocaproic acid	0.93	High	NS	High
Hydroxyphenyl pyruvic acid	5.85	Low	NS	Low
Indoxyl sulfate	7.20	Low	NS	Low
Isopropyl alcohol	4.00	NS	NS	Low
Lactic acid	1.34	High	High	High
Leucine	1.70	High	NS	High

Mandelic acid	7.38	Low	NS	Low
Mannitol	3.87	High	NS	High
Methionine	2.12	High	NS	High
Methylguanidine	3.37	Low	Low	Low
Myo-inositol	3.63	High	High	NS
N6-acetyl-L-lysine	1.88	High	NS	High
N-Acetylglutamic acid	2.02	High	NS	High
N-acetyl-L-alanine	2.00	High	NS	High
N-Methyl-a-aminoisobutyric acid	3.84	High	NS	High
Oxaloacetic acid	2.36	High	High	NS
Oxoglutaric Acid	2.98	High	High	NS
Phenylacetylglycine	7.41	High	NS	High
Phosphoenolpyruvic acid	5.31	High	High	NS
Propionic acid	1.04	High	NS	High
Putrescine	3.04	Low	NS	Low
Pyridoxal	2.49	Low	NS	Low
Pyridoxamine 5'-phosphate	4.84	Low	NS	Low
Quinolinic acid	7.43	Low	NS	Low
Sarcosine	2.71	Low	NS	Low
Serine	3.95	Low	Low	NS
Sorbitol	3.78	NS	High	Low
Suberic acid	2.16	Low	NS	Low
Succinic acid	2.41	High	NS	High
Taurine	3.40	High	NS	High
Theophylline	3.33	NS	Low	High
Trimethylamine oxide	3.25	High	NS	High

Tyrosine	6.90	Low	NS	Low
Valine	1.008	Low	NS	Low

NS – Not significant
