# Supplementary material for: Plasma metabolomic study in perinatally HIV-infected children using 1H NMR spectroscopy reveals perturbed metabolites that sustain during therapy
Source: PLoS One. 2020 Aug 31;15(8):e0238316. doi: 10.1371/journal.pone.0238316 (PMC7458310; doi:10.1371/journal.pone.0238316)
Supplement: S2 Table — (PDF) [file pone.0238316.s003.pdf]

S2 Table. Perturbed metabolic pathways between treatment naïve, ART-suppressed HIV infected children and uninfected controls

Pathway name	Total compounds	p value	Hits	Name of the Hits
Glycine, serine and threonine metabolism	48	1.17E-06	9	Serine, Choline, Glyceric acid, dimethylglycine, Glycine, Aspartic acid, Sarcosine, Creatine, 2-Ketobutyric acid
Aminoacyl-tRNA biosynthesis	75	5.30E-05	9	Glutamate, Glycine, Aspartic acid, Serine, Methionine, Valine, Alanine, Leucine, Tyrosine
Propanoate metabolism	35	9.85E-07	8	2-Ketobutyric acid, Propionic acid, Succinic acid, Lactic acid, Isopropyl alcohol, Acetoacetic acid, Valine, Acetone
Alanine, aspartate and glutamate metabolism	24	8.21E-07	7	N-Acetylaspartic acid, Aspartic acid, Alanine, Oxoglutaric acid, Glutamine, Oxaloacetic acid, Succinic acid
Glyoxylate and dicarboxylate metabolism	50	0.000145	7	cis-Aconitic acid, Oxoglutaric acid, Formic acid, Citric acid, Glyceric acid, Oxaloacetic acid, Succinic acid
Citrate cycle (TCA cycle)	20	4.50E-06	6	Succinic acid, Oxoglutaric acid, Oxaloacetic acid, cis-Aconitic acid, Citric acid, Phosphoenolpyruvic acid
Glycolysis or Gluconeogenesis	31	6.95E-05	6	Ethanol, Lactic acid, Phosphoenolpyruvic acid, Glucose, Oxaloacetic acid, Acetic acid
Nitrogen metabolism	39	0.000265	6	Tyrosine, Taurine, Aspartic acid, Glutamine, Glycine, Formic acid
Pyruvate metabolism	32	0.000835	5	Phosphoenolpyruvic acid, Oxaloacetic acid, Lactic acid, Formic acid, Acetic acid
Cysteine and methionine metabolism	56	0.010186	5	Serine, Methionine, Aspartic acid, 2-Ketobutyric acid, Alanine

Tyrosine metabolism	76	0.0342	5	3,4-Dihydroxymandelate, Tyrosine, Hydroxyphenylpyruvic acid, Acetoacetic acid, Succinic acid
Arginine and proline metabolism	77	0.035916	5	Glutamine, Aspartic acid, N-Acetyl-L-alanine, Creatine, Sarcosine
Vitamin B6 metabolism	32	0.006601	4	Pyridoxamine 5'-phosphate, Oxoglutaric acid, Pyridoxal, 4-Pyridoxic acid
Pentose phosphate pathway	32	0.006601	4	6-Phosphogluconic acid, Glucono-1,5-lactone, Glucose, Glyceric acid
Methane metabolism	34	0.008214	4	Glycine, Formic acid, Trimethylamine N-oxide, Serine
Butanoate metabolism	40	0.014542	4	Beta-Hydroxybutyrate, Acetoacetic acid, Succinic acid, Oxoglutaric acid
Synthesis and degradation of ketone bodies	6	0.000252	3	Acetoacetic acid, Beta-Hydroxybutyrate, Acetone
Cyanoamino acid metabolism	16	0.005951	3	Aspartic acid, Glycine, Serine
Taurine and hypotaurine metabolism	20	0.011314	3	Taurine, Alanine, Acetic acid
Phenylalanine, tyrosine and tryptophan biosynthesis	27	0.025773	3	Tyrosine, Phosphoenolpyruvic acid, Hydroxyphenylpyruvic acid
Valine, leucine and isoleucine biosynthesis	27	0.025773	3	Leucine, Valine, 2-Ketobutyric acid

D-Glutamine and D-glutamate metabolism	11	0.0273	2	Glutamine, Oxoglutaric acid
