# Supplementary figures and images for: Plasma metabolomic study in perinatally HIV-infected children using 1H NMR spectroscopy reveals perturbed metabolites that sustain during therapy
Source: PLoS One. 2020 Aug 31;15(8):e0238316. doi: 10.1371/journal.pone.0238316 (PMC7458310; doi:10.1371/journal.pone.0238316)

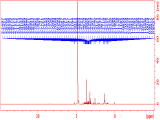

Supplement: S1 Raw data — (TGZ) [file pone.0238316.s004.tgz › Supplementary_raw_data/Controls/C11/pdata/1/thumb.png]

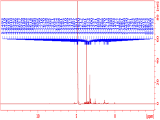

Supplement: S1 Raw data — (TGZ) [file pone.0238316.s004.tgz › Supplementary_raw_data/Controls/C2/pdata/1/thumb.png]

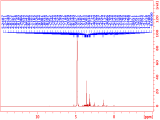

Supplement: S1 Raw data — (TGZ) [file pone.0238316.s004.tgz › Supplementary_raw_data/Controls/C5/pdata/1/thumb.png]

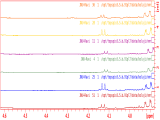

Supplement: S1 Raw data — (TGZ) [file pone.0238316.s004.tgz › Supplementary_raw_data/Controls/C10/pdata/1/thumb.png]

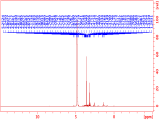

Supplement: S1 Raw data — (TGZ) [file pone.0238316.s004.tgz › Supplementary_raw_data/Controls/C4/pdata/1/thumb.png]

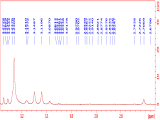

Supplement: S1 Raw data — (TGZ) [file pone.0238316.s004.tgz › Supplementary_raw_data/Controls/C3/pdata/1/thumb.png]

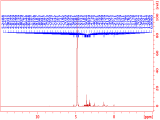

Supplement: S1 Raw data — (TGZ) [file pone.0238316.s004.tgz › Supplementary_raw_data/Controls/C6/pdata/1/thumb.png]

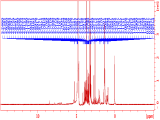

Supplement: S1 Raw data — (TGZ) [file pone.0238316.s004.tgz › Supplementary_raw_data/Controls/C1/pdata/1/thumb.png]

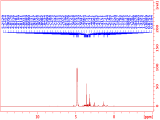

Supplement: S1 Raw data — (TGZ) [file pone.0238316.s004.tgz › Supplementary_raw_data/Controls/C8/pdata/1/thumb.png]
